# COVID-19 and Unplanned Paediatric Hospital Readmissions in Australia: A Retrospective Cohort Study

**DOI:** 10.3390/ijerph23070942

**Published:** 2026-07-22

**Authors:** Huma Sadulla, Adhya Miriam Tom, Gary Low, Anthony Liu, Habib Bhurawala

**Affiliations:** 1 School of Medicine, Western Sydney University, Sydney, NSW 2560, Australia; huma.sadulla@health.nsw.gov.au; 2 College of Medicine, Gulf Medical University, Ajman P.O. Box 4184, United Arab Emirates; 2021m057@mygmu.ac.ae; 3 Nepean Clinical School, Faculty of Medicine and Health, University of Sydney, Sydney, NSW 2050, Australia; gary.low@health.nsw.gov.au (G.L.); anthony.liu@sydney.edu.au (A.L.); 4 Research Directorate, Nepean Hospital, Nepean Blue Mountains Local Health District, Kingswood, NSW 2747, Australia; 5 Department of Paediatrics, Nepean Hospital, Nepean Blue Mountains Local Health District, Kingswood, NSW 2747, Australia; 6 Faculty of Medicine, Notre Dame University, Sydney, NSW 2007, Australia

**Keywords:** COVID-19, hospital readmission, child, infant, retrospective studies, Australia

## Abstract

**Highlights:**

**Public health relevance—How does this work relate to a public health issue?**

- Unplanned paediatric hospital readmissions are an important public health and healthcare quality indicator, reflecting challenges in disease management, discharge planning, and continuity of care.
- This study examines how a major public health disruption, the COVID-19 pandemic, influenced paediatric healthcare utilisation and readmission patterns in an Australian hospital setting.

**Public health significance—Why is this work of significance to public health?**

- This study demonstrates how major system-wide disruptions such as the COVID-19 pandemic can significantly alter paediatric healthcare utilisation patterns, highlighting the importance of resilient health systems and adaptive discharge and follow-up pathways to maintain continuity of care during public health crises.
- By identifying consistently high-risk groups for unplanned readmissions (infants, males, and socio-economically patterned populations), the findings provide actionable evidence to guide targeted preventive interventions, reduce avoidable hospital use, and improve equity in paediatric health service delivery.

**Public health implications—What are the key implications or messages for practitioners, policy makers and/or researchers in public health?**

- The observed reduction in paediatric readmissions during the COVID-19 period highlights how system-level changes (e.g., reduced healthcare utilisation, altered admission thresholds, and increased reliance on telehealth) can substantially influence hospital demand, underscoring the need for policy frameworks that maintain safe access to care even during major public health disruptions.
- Persistent disparities in readmission risk among infants, males, and socio-demographically patterned populations indicate the need for targeted, equity-focused discharge planning, strengthened primary care follow-up, and data-driven risk stratification tools to support clinicians and inform preventive public health strategies.

**Abstract:**

**Background**: Unplanned paediatric hospital readmissions are key indicators of healthcare quality, often reflecting issues in discharge planning and continuity of care. The COVID-19 pandemic significantly disrupted healthcare delivery and utilisation, yet its impact on paediatric readmission patterns in Australia remains unclear. **Methods**: A 5-year single-centre retrospective cohort study of 21,262 paediatric admissions and unplanned readmissions at a non-tertiary metropolitan Australian hospital between 21 March 2017 and 20 March 2023 was conducted. Admissions up to 20 March 2020 were classified as pre-pandemic, and those from 21 March 2020 as during the COVID-19 pandemic. We analysed patient characteristics, including gender, age, First Nations status, primary diagnosis, and socio-economic status, to identify UHR trends. **Results**: During the COVID-19 period, total paediatric admissions declined by nearly 30%, and the readmission rate decreased from 7.5% to 4.6% compared with the pre-pandemic period. Across both cohorts, higher readmission rates were observed among male patients, infants aged 0–12 months, and children from more socio-economically advantaged areas. Readmissions among First Nations children and those with communicable diseases declined during the pandemic period. **Conclusions**: A higher proportion of males and patients under 12 months of age had higher readmission rates across both periods. These findings highlight the need for targeted early intervention, age-specific discharge planning, and strengthened follow-up care for high-risk groups. Further research should extend the study period beyond the pandemic and include larger cohorts to better characterise long-term trends and improve generalisability.

## 1. Introduction

According to the Australian Public Hospital Performance Indicator 23 (PI 23) framework, patients readmitted to a hospital within a specified period after discharge are classified as unplanned hospital readmissions (UHRs) [1].

High UHR rates can indicate potential disease exacerbations, insufficient inpatient management, poor discharge planning or inadequate follow-up, all of which can negatively impact patient outcomes [2]. UHRs also induce social and financial stress on patients and their families, affecting their quality of life [3].

Insights into the patterns of UHRs can help identify potential areas for intervention to lower these rates and improve patient outcomes while also reducing the costs to the healthcare system [4]. Studies have utilised these patterns to develop predictive computer modelling techniques that can identify patients with a higher likelihood of unplanned readmissions to implement early intervention [5,6].

Numerous paediatric studies have analysed patient characteristics, including age, gender, cause of admission, comorbidities, health insurance, and length of stay (LOS), to determine trends for UHRs [7]. However, Australian literature on paediatric UHR trends remains limited. Pre-existing studies primarily focus on specific health conditions or mental illness [8]. The broader analyses include a 2019 study on 30-day all-cause readmission risk factors [9] and a five-year retrospective cohort study of unplanned readmissions [10], but no research has assessed post-COVID-19 changes in UHR trends.

The COVID-19 pandemic represented a major systemic shock to healthcare delivery, disrupting access to acute and preventive services and challenging the resilience of health systems globally. In paediatric populations, service disruptions, altered care-seeking behaviour, and rapid implementation of telehealth models may have substantially modified patterns of hospital utilisation. Understanding how such system-wide changes affect unplanned hospital readmissions provides important insight into health system performance, continuity of care, and equity of access during periods of crisis. Accordingly, this study aimed to describe changes in admission-level utilisation patterns before and during the COVID-19 pandemic rather than identify independent predictors of readmission.

## 2. Method

### 2.1. Study Design and Setting

This was a 5-year retrospective cohort study conducted at a major non-tertiary metropolitan hospital in Australia. The study was reported in accordance with the Strengthening the Reporting of Observational Studies in Epidemiology (STROBE) guidelines [11].

### 2.2. Data Source and Study Population

Data were extracted from the hospital’s electronic medical record system for all paediatric patients aged under 16 years admitted between 21 March 2017 and 20 March 2023. A total of 21,262 patient records were identified and included in the final analysis after data cleaning and verification. The unit of analysis was the hospital admission episode rather than the individual patient. Consequently, a child could contribute more than one admission during the study period. If a patient experienced multiple unplanned readmissions within separate admission episodes, each eligible readmission was included as an independent hospitalisation event in accordance with the study objective of evaluating hospital utilisation patterns. Record accuracy was ensured by cross-checking extracted data against individual patient files using unique medical record numbers.

### 2.3. Definition of Exposure Period

The study period was divided into two phases based on the onset of the COVID-19 pandemic in Australia. The date of 21 March 2020 (Australian international border closure) [12] was used as the cut-off point to define the pre-pandemic period (21 March 2017–20 March 2020) and the COVID-19 pandemic period (21 March 2020–20 March 2023).

### 2.4. Outcome Definition

The primary outcome was a 28-day unplanned hospital readmission. An index admission was defined as an eligible unplanned paediatric hospital admission episode occurring during the study period that resulted in discharge. Index admissions were not restricted to a patient’s first-ever hospitalisation or first admission during the study period; therefore, a patient could contribute multiple index admission episodes if they had separate eligible admissions. Each admission episode was evaluated sequentially to determine whether it resulted in a subsequent unplanned readmission within 28 days. Each admission episode was assessed sequentially based on admission and discharge dates to determine readmission status. Planned admissions, including elective procedures, scheduled investigations, and other pre-arranged hospitalisations, were excluded from readmission classification.

### 2.5. Inclusion and Exclusion Criteria

All paediatric admissions during the study period were eligible for inclusion. Patients aged 16 years and older were excluded. Planned or elective admissions occurring within 28 days of a previous admission were excluded to ensure accurate identification of unplanned readmissions. Records with missing key demographic variables (*n* = 55) were excluded from the analysis. No imputation methods were applied, as the proportion of missing data was small and limited to key demographic variables. After exclusion of these records, all included admissions had complete data for the variables analysed. Elective admissions were identified using hospital admission type coding within the electronic medical record system and excluded prior to analysis. Admissions classified as planned, scheduled, or elective were not considered index admissions or readmissions. Emergency and other non-elective admissions occurring within 28 days of discharge from a previous eligible admission were classified as unplanned readmissions.

### 2.6. Variables and Measures

The primary exposure variable was admission period (pre-pandemic vs. COVID-19 pandemic). Covariates included age, sex, First Nations status, primary diagnosis, and socio-economic status. Socio-economic status was determined using the Index of Relative Socio-economic Advantage and Disadvantage (IRSAD) deciles based on patient postcode [13].

### 2.7. Data Management and Bias Considerations

Potential sources of bias included changes in healthcare-seeking behaviour during the COVID-19 pandemic, possible misclassification of readmissions, and incomplete capture of readmissions occurring outside the study hospital. Although all admissions within the hospital were captured through a single integrated electronic medical record system, children who sought care at another hospital during the study period would not have been identified. This limitation may have resulted in underestimation of readmission rates, particularly during the COVID-19 pandemic when healthcare access pathways, parental care-seeking behaviour, and hospital utilisation patterns were altered.

### 2.8. Ethical Approval

Ethical approval was obtained from the Human Research Ethics Committee of Nepean Blue Mountains Local Health District (ETH00646). A waiver of informed consent was granted due to the retrospective nature of the study and use of de-identified routinely collected data.

### 2.9. Statistical Analysis

All statistical analyses were conducted using R Studio (version 4.5.2) [14]. Descriptive statistics were used to summarise patient characteristics, with categorical variables reported as frequencies and percentages.

As the objective was to examine admission-level utilisation patterns, analyses were performed at the hospitalisation level rather than the patient level. Repeated admissions by the same patient were retained in the dataset if they fulfilled the study definition of an eligible admission or readmission.

Stratified analyses were performed to examine distributions of admissions and readmissions according to sex, First Nations status, socio-economic status, and primary diagnosis. Age-specific trends were visualised using line plots, with age categories selected to reflect clinically relevant paediatric subgroups, particularly infancy, which is known to be associated with increased readmission risk.

As the primary objective of this study was to describe changes in admission and readmission patterns before and during the COVID-19 pandemic, analyses were prespecified to be descriptive. Multivariable regression modelling was not undertaken because the study was not designed to identify independent predictors of readmission or estimate adjusted associations. As all readmissions were captured within the hospital electronic medical record system, loss to follow-up was not applicable.

## 3. Results

**Figure 1:**
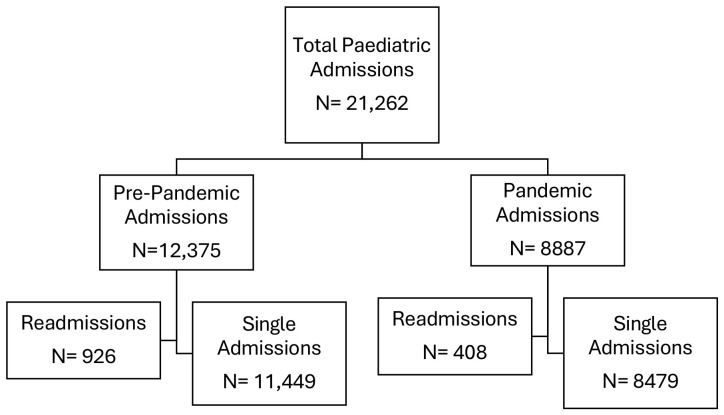
Flow diagram of total paediatric admissions and number of single admissions and readmissions in both cohorts.

There were 12,375 paediatric admissions, including 926 readmissions, during the pre-pandemic period (21 March 2017–20 March 2020). In the COVID-19 pandemic period (21st March 2020–20th March 2023), there were 8887 admissions, with 408 readmissions recorded (Figure 1).

Table 1 compares the demographic characteristics of paediatric single-admission episodes between the pre-pandemic and COVID-19 pandemic periods. The number of single admissions decreased from 11,449 before the pandemic to 8479 during the pandemic, reflecting an overall reduction in paediatric hospitalisations. The sex distribution remained stable across both periods, with male patients accounting for 57.0% of single admissions before the pandemic and 56.6% during the pandemic. Similarly, the proportion of First Nations children changed only marginally, from 11.0% before the pandemic to 11.4% during the pandemic. The distribution of admissions across IRSAD deciles was also largely comparable between periods, with the greatest proportion of admissions occurring among children residing in areas classified as decile 6 (34.0% pre-pandemic vs. 35.9% during the pandemic), followed by deciles 9 and 5. Overall, the demographic profile of paediatric single admissions remained consistent despite the substantial reduction in the total number of hospital admissions during the COVID-19 pandemic.

Table 2 compares the demographic characteristics of paediatric 28-day unplanned readmission episodes before and during the COVID-19 pandemic. The number of readmissions declined from 926 (7.5% of all admissions) in the pre-pandemic period to 408 (4.6%) during the pandemic. Male patients represented the majority of readmissions in both periods, although their proportion decreased from 58.5% before the pandemic to 53.9% during the pandemic, with a corresponding increase among females (41.5% to 46.1%). The proportion of readmissions among First Nations children also declined slightly, from 15.3% before the pandemic to 13.5% during the pandemic. Across both periods, the highest proportion of readmissions occurred among children residing in IRSAD decile 6 areas (35.4% pre-pandemic and 39.5% during the pandemic), followed by deciles 5 and 9. Overall, while the total number of readmissions decreased during the COVID-19 pandemic, the demographic distribution of readmitted children remained broadly similar between the two periods, with only modest changes in sex, First Nations status, and socioeconomic distributions.

**Figure 2:**
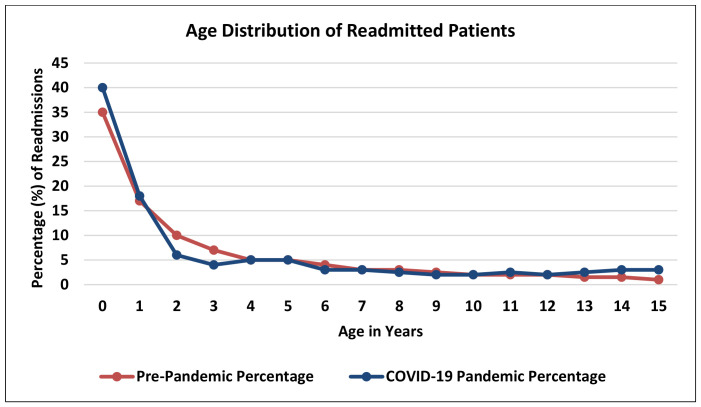
Age Distribution of Readmitted Patients in both cohorts.

The age distribution of readmitted patients from before and during the COVID-19 pandemic remained notably consistent (Figure 2). The highest incidence of readmissions was observed among patients under 1 year of age, who comprised 36.2% of the pre-pandemic group and 40.4% of the COVID-19 pandemic cohort.

**Figure 3:**
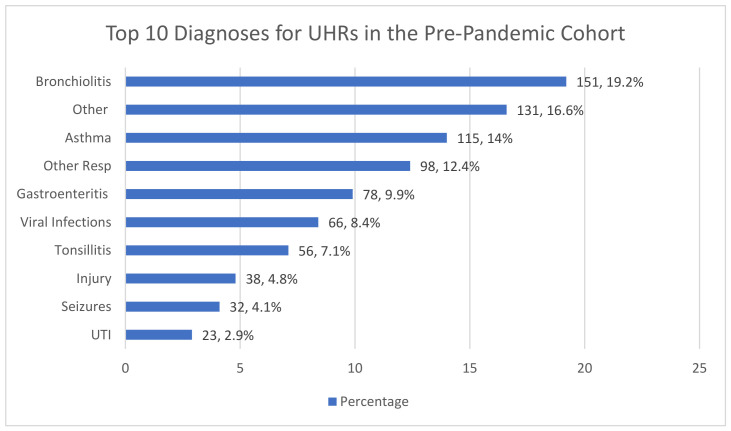
Top 10 Diagnoses for UHRs in the Pre-Pandemic Cohort.

**Figure 4:**
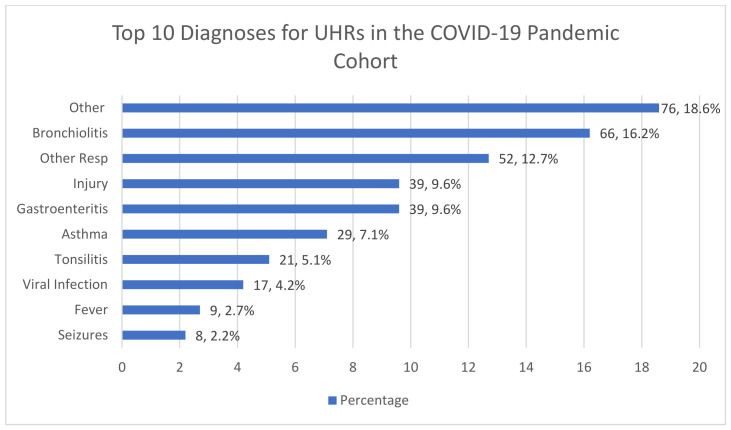
Top 10 Diagnoses for UHRs in the COVID-19 Pandemic Cohort.

As shown in Figure 3 and Figure 4, nine of the ten most prevalent diagnoses for readmissions remain unchanged in both the pre-pandemic and COVID-19 pandemic groups. The category of ‘Other’ diagnoses, such constipation gastroesophageal reflux disease, nose and nasal sinus disorders, and ‘Other Resp’, which included breathing abnormalities, volume depletion and nasal disorders, were not prevalent enough for individual analysis. Socio-economic distribution across readmitted patients in both periods showed a slight skew towards socio-economic advantage (deciles 6–10). In the pre-pandemic cohort, 61.7% of readmitted patients were classified in the 6th to 10th decile, indicating higher socio-economic advantage; during the COVID-19 pandemic, this proportion increased to 69%.

## 4. Discussion

Our findings of reduced paediatric UHRs can be correlated with the decline in usage of healthcare services between March and June 2020 compared to the same period in 2019, as inpatient admissions in public hospitals decreased by 14.3%, ambulance incidents decreased by over 7% and emergency department (ED) presentation decreased by almost 14% [15].

Additional international studies have found that parents were increasingly reluctant to take sick children to the hospital for fear of contracting the COVID-19 virus. However, although the number of presentations decreased, disease severity at presentation increased due to delayed access to medical care and delayed diagnosis [16]. Although lower admission and readmission rates generally benefit the healthcare system by reducing demand on resources, in this context, they may mask underlying disease progression. The observed decline in admissions and readmissions during the pandemic likely reflects widespread care avoidance [17] due to fear of COVID-19 exposure. Such avoidance may ultimately lead to worse health outcomes and higher healthcare costs, as untreated or delayed conditions progress and require more intensive and costly interventions. In addition to changes in patient behaviour, system-level factors such as modified admission thresholds, increased use of telehealth, and altered models of care during the COVID-19 pandemic may also have contributed to the observed reduction in readmissions.

### 4.1. Gender

In our study, males consistently had higher readmission rates across both cohorts. Another Australian study of unplanned paediatric admissions also found that 55.6% of readmitted children were male [10]. Other studies that have investigated predictors for detecting children at higher risk of readmission have identified that while male children are at increased risk of readmission in cohorts, they are the dominant gender for total admissions; they remain the higher readmission group in cohorts even while females make up the greater proportion of the admission pool [18]. This suggests that the trend in readmissions by gender remained largely unchanged from before and during the COVID-19 pandemic.

### 4.2. First Nations Status

Our study found that readmission rates among First Nations children were lower during the COVID-19 pandemic than before. Although this reduction was not massive, it represents an encouraging trend that warrants further investigation, given the historically disproportionate health disadvantages experienced by First Nations patients [19]. One Australian study found that 70% of children in a rural Indigenous community were admitted at least once before the age of 7, with infants accounting for over a third of admissions [20].

No Australian-specific studies have evaluated the impact of the COVID-19 pandemic on paediatric readmission patterns among First Nations children. In our cohort, First Nations children demonstrated a lower proportion of readmissions during the pandemic period compared with the pre-pandemic period. However, this finding should be interpreted cautiously, as our study did not evaluate changes in healthcare access, service utilisation, community-based interventions, or other factors that may have influenced readmission patterns during this period. Further research incorporating patient-level clinical, social, and healthcare access data is required to better understand the factors underlying these observed differences.

### 4.3. Patient Age

A stable trend was observed across both the pre-pandemic and COVID-19 paediatric cohorts in the age distribution of readmitted patients. The highest readmission rates occurred in infants under 1 year of age, followed by those aged 1 year, with rates declining with increasing age. An Australian retrospective study of infants under three months found that age ≤ 21 days was the most significant predictor of readmission [21]. This may relate to unestablished feeding patterns before discharge, which have been shown to increase the likelihood of neonatal readmissions [22].

Conversely, a study of 30-day all-cause unplanned paediatric readmissions reported that patients older than 13 years were 1.5 times more likely to be readmitted than younger children [6]. Differences in healthcare access, hospital admission criteria, or chronic disease prevalence among adolescents may account for this discrepancy.

Higher proportions of readmissions were observed among younger children. This likely reflects their immature immune systems, higher susceptibility to infection, and the challenges of managing complex medical conditions in early infancy, all of which can increase the risk of post-discharge complications [23]. The persistence of this pattern across both periods underscores the need for age-specific discharge planning and follow-up strategies.

### 4.4. Primary Diagnoses

The top ten diagnoses leading to readmissions account for approximately 66% of the total in both cohorts. Among these, nine of the ten most common diagnoses for unplanned readmissions remained consistent between the pre-pandemic and COVID-19 pandemic periods. A notable reduction in readmissions related to respiratory and viral infections was observed during the COVID-19 pandemic, with bronchiolitis no longer the predominant primary diagnosis for readmission. This trend likely reflects the impact of public health measures such as mask-wearing, social distancing, stringent hygiene practices and isolation during lockdown.

Similar trends in paediatric admissions for communicable diseases were also observed in the Netherlands during the COVID-19 pandemic [17]. While these results are reassuring regarding the decline of viral infection spread in the paediatric population, there is still a need for ongoing monitoring in case of resurgence as public health measures relax over time.

### 4.5. Socio-Economic Status (SES)

Patients’ socio-economic status in this study was determined using the IRSAD score along with their area of residence. The IRSAD score provides a relative measure of the socio-economic standing of patients’ localities compared with the wider Australian community [13]. This helps to provide information regarding their access to resources, health disparities, and the other social determinants of health that impact their overall health outcomes. Lower IRSAD scores correspond to greater socio-economic disadvantage [13]. This study found that, in both the pre-pandemic and COVID-19 pandemic cohorts, a greater proportion of readmission episodes occurred among children residing in areas with higher IRSAD scores. This finding reflects area-level socioeconomic patterns and should not be interpreted as indicating that individual patients or families had greater socioeconomic advantage. The Western Australian study also found higher rates of UHRs in patients with a high IRSAD score [9]. In contrast, a recent American study has found that patients from high-poverty neighbourhoods were 24% more likely to be readmitted than others [24]. This deviation in findings between Australian and non-Australian studies suggests that the impact of socio-economic status on readmission rates varies across different healthcare system dynamics. Families from more advantaged areas may be more likely to seek hospital care post-discharge due to better access to healthcare, a lower threshold for concern, or higher health literacy. This potential confounder should be explored in future research. Overall, while socio-economic disadvantage is typically associated with higher healthcare needs and greater access barriers, these findings highlight the need to explore why readmission rates appear higher among more advantaged populations in Australia.

However, the interpretation of IRSAD-based socioeconomic patterns should be approached with caution. IRSAD is an area-level measure derived from residential postcode and reflects the relative socioeconomic characteristics of geographic areas rather than individual-level socioeconomic circumstances. In a single-hospital catchment population, the distribution of IRSAD deciles may be influenced by the geographic composition of the hospital’s referral area, with some deciles represented by a limited number of residential areas. Therefore, the observed distribution across IRSAD deciles may not reflect a true population-wide socioeconomic gradient and should not be interpreted as individual socioeconomic advantage or disadvantage. Future multicentre studies incorporating individual-level socioeconomic indicators, such as household income, parental education, employment status, or health insurance status, may provide a more comprehensive assessment of socioeconomic influences on paediatric readmissions.

Limitations of this study include its relatively short time frame, with only three years of COVID-19 pandemic paediatric admissions data available for analysis. Another limitation is that patient-level clinical factors that may influence readmission risk, including chronic medical conditions, complexity of underlying disease, previous hospitalisation history, and healthcare utilisation patterns before the index admission, were not available for adjustment in the current analysis. Children with complex chronic conditions may contribute disproportionately to repeated admissions, and the admission-level approach may therefore overrepresent patients with higher healthcare needs. Additionally, multivariate analysis was not conducted. This study was based in a single non-tertiary paediatric unit in Australia, and the findings may not apply to other populations, especially in different healthcare contexts globally.

Future research should extend the COVID-19 pandemic study period to yield long-term insights, and larger, multicentre study cohorts from other Australian paediatric units should be considered to enhance the reliability of the findings.

## 5. Conclusions

During the COVID-19 pandemic, overall readmission rates declined, with descriptive reductions observed among First Nations children and communicable disease-related admissions. However, these findings require further investigation to determine the underlying factors contributing to these patterns. Pre-existing trends persisted, including higher readmission rates among males, infants, and socio-economically advantaged patients. These findings highlight the need to strengthen health system resilience, continuity of care, and equity in access during periods of healthcare disruption, such as improved discharge planning, structured parental education, and coordinated post-discharge follow-up protocols, to reduce the likelihood of unplanned hospital readmissions.

## Figures and Tables

**Table 1:** Demographic characteristics of paediatric single-admission episodes before and during the COVID-19 pandemic.

Variable	Pre-Pandemic Single Admissions *n* (%)	Pandemic Single Admissions *n* (%)
Total	11,449 (92.5)	8479 (95.4)
Gender		
Male	6530 (57.0)	4797 (56.6)
Female	4919 (43.0)	3682 (43.4)
First Nations status		
Yes	1256 (11.0)	967 (11.4)
No	10,193 (89.0)	7512 (88.6)
IRSAD decile		
1	581 (5.1)	330 (3.9)
2	133 (1.2)	133 (1.6)
3	1057 (9.3)	691 (8.2)
4	92 (0.8)	46 (0.5)
5	2080 (18.2)	1438 (17.0)
6	3884 (34.0)	3038 (35.9)
7	636 (5.6)	501 (5.9)
8	370 (3.2)	339 (4.0)
9	2244 (19.7)	1640 (19.4)
10	339 (3.0)	305 (3.6)

**Table 2:** Demographic characteristics of paediatric 28-day unplanned readmission episodes before and during the COVID-19 pandemic.

Variable	Pre-Pandemic Readmissions *n* (%)	Pandemic Readmissions *n* (%)
Total	926 (7.5)	408 (4.6)
Gender		
Male	542 (58.5)	220 (53.9)
Female	384 (41.5)	188 (46.1)
First Nations status		
Yes	142 (15.3)	55 (13.5)
No	784 (84.7)	353 (86.5)
IRSAD decile		
1	40 (4.3)	11 (2.7)
2	4 (0.4)	1 (0.2)
3	109 (11.8)	32 (7.8)
4	6 (0.7)	1 (0.2)
5	195 (21.1)	81 (19.9)
6	327 (35.4)	161 (39.5)
7	35 (3.8)	17 (4.2)
8	22 (2.4)	17 (4.2)
9	172 (18.6)	76 (18.6)
10	13 (1.4)	10 (2.5)

## Data Availability

The data presented in this study are not publicly available due to ethical and privacy restrictions. De-identified datasets may be made available from the corresponding author upon reasonable request and subject to approval by the Human Research Ethics Committee of Nepean Blue Mountains Local Health District.
